# From Fully Physical to Virtual Sensing for Water Quality Assessment: A Comprehensive Review of the Relevant State-of-the-Art

**DOI:** 10.3390/s21216971

**Published:** 2021-10-20

**Authors:** Thulane Paepae, Pitshou N. Bokoro, Kyandoghere Kyamakya

**Affiliations:** 1 Department of Mathematics and Applied Mathematics, University of Johannesburg, Doornfontein 2028, South Africa; tpaepae@uj.ac.za; 2 Department of Electrical and Electronic Engineering Technology, University of Johannesburg, Doornfontein 2028, South Africa; 3 Institute for Smart Systems Technologies, Transportation Informatics Group, Alpen-Adria Universität Klagenfurt, 9020 Klagenfurt, Austria; kyandoghere.kyamakya@aau.at

**Keywords:** review, soft sensor, physical sensor, irrigation water quality parameters, low cost, specification book, measurement accuracy, machine learning, deep learning, internet of things

## Abstract

Rapid urbanization, industrial development, and climate change have resulted in water pollution and in the quality deterioration of surface and groundwater at an alarming rate, deeming its quick, accurate, and inexpensive detection imperative. Despite the latest developments in sensor technologies, real-time determination of certain parameters is not easy or uneconomical. In such cases, the use of data-derived virtual sensors can be an effective alternative. In this paper, the feasibility of virtual sensing for water quality assessment is reviewed. The review focuses on the overview of key water quality parameters for a particular use case and the development of the corresponding cost estimates for their monitoring. The review further evaluates the current state-of-the-art in terms of the modeling approaches used, parameters studied, and whether the inputs were pre-processed by interrogating relevant literature published between 2001 and 2021. The review identified artificial neural networks, random forest, and multiple linear regression as dominant machine learning techniques used for developing inferential models. The survey also highlights the need for a comprehensive virtual sensing system in an internet of things environment. Thus, the review formulates the specification book for the advanced water quality assessment process (that involves a virtual sensing module) that can enable near real-time monitoring of water quality.

## 1. Introduction

### 1.1. Background and Motivation

Water is an essential resource for every aspect of human and ecosystem health and survival. Apart from domestic uses, water is required for manufacturing processes, agricultural production, hydroelectric power generation, assimilation of waste, conservation (or enhancement of wildlife and fish), and for various other purposes. On the other end, change drivers such as emerging contaminants, climate change, the substantial growth of human populations, rural–urban migration, variation of pollutant discharge patterns, human activities, and lack of adequate sanitation facilities (particularly in developing countries) have resulted in the deterioration of surface (dams, lakes, and rivers) and groundwater quality at an alarming rate [1,2].

The term water quality (WQ) refers to the biological, chemical, and physical characteristics (or properties) of a water supply system that will influence its fitness for a particular use [3]. Most of these properties are influenced or controlled by changes in constituents (or parameters) which are either suspended or dissolved in water [4]. Therefore, monitoring water quality involves a regular detection of these characteristic parameters at specific locations to provide data (or information) to define current conditions or establish trends [5,6]. On the other hand, WQ assessment refers to the *overall process* of evaluating the biological, chemical, and physical nature of water concerning the human effects and the envisioned water application [7]. Interpretation and reporting of results are the critical aspects of an assessment of monitoring and recommending future actions [7]. Generally, there is a logical sequence comprising water quality monitoring (WQM), followed by assessment, and then by management [7]. Institutional responsibilities for WQM in most countries are established by national guidelines. Normally, these responsibilities are categorized into: (i) operational monitoring, which serves to inform corrective action or decision-making concerning control measures (e.g., water treatment, source water protection) by water utilities; and (ii) compliance monitoring by an independent third party (or agency), usually with a specific mandate for public health protection [8].

WQM relies on the traditional laboratory-based approach, where trained field personnel travel to a water source, collect water samples, preserve them, and then transport them to accredited laboratories for subsequent analysis [9,10]. Factors such as the laboratory process accuracy, precision, and adherence to standard techniques for regulatory and legal procedures have maintained the need for this traditional methodology [9,10,11]. Although this conventional method is still dominant in most developing countries, it has several drawbacks [10,12]:

(i) Developing state-of-the-art laboratory facilities and their subsequent maintenance is expensive;
(ii) It generally requires specialized equipment and expert personnel to assess WQ;
(iii) Results can be questionable because of the field-sampling error and errors introduced by the malfunction or miscalibration of laboratory equipment;
(iv) It lacks real-time WQ information and therefore prone to time-delayed responses to pollution incidences;
(v) It is labour-intensive and time-consuming.

To overcome these limitations, new monitoring technologies were introduced around the 1880s, where the first thermostat to emerge in 1883 is considered to be the first modern sensor [13]. For WQM, the first sensor to appear (around 1930) was the glass pH electrode, which appeared along with a pH meter [12]. This was followed by extensive research on the development of potable (or hardware) sensors for other WQ parameters such as dissolved oxygen, turbidity, water temperature, chlorophyll-a, electrical conductivity, ammonia nitrogen, and salinity, resulting in the establishment of relatively mature and low-cost sensors for these parameters [14]. Though this developmental act somewhat improved the traditional method, most of the issues mentioned above were still not addressed. For instance, such schemes could still not provide real-time information about WQ; the spatial coverage was inadequate since data collection was still manual, transportation delays were still a factor, etc. [15].

With more advances in portable sensors, computing technologies, and communication, the research moved towards using wireless sensor network (WSN) technology. Generally, WSN-based WQM systems work as follows [15,16]: first, a WSN comprises sensors that take specific parameter readings (e.g., pH) that are then sent to a controller through a wireless (optional) communication device. Next, the information is transmitted to the central station through wireless communication media, where all the necessary analysis and processing takes place. The results are finally communicated to relevant water authorities.

While WSN significantly resolved some drawbacks of previous systems, they also have some limitations. For example, high power requirements, the vulnerability of sensor data to cyber-attacks, high installation or maintenance costs, storage issues, etc. [3,16]. At the same time, researchers also involved machine learning (ML) techniques in assessing WQ using a relatively small number of WQ parameters [15]. Undoubtedly, this significantly enhanced the effectiveness of WSN systems. Nonetheless, critical issues such as data privacy and security and data processing and storage were not fully resolved [17].

To this effect, development in research resulted in the application of internet of things (IoT) technology. Using IoT enables real-time monitoring of water from anywhere through a combination of internet services, portable sensors, communication media, and digital computing devices [18]. It is important to note that WSN is a major technology enabling the IoT [19], and this integration allows the WSN to reach its full potential [17].

Despite these advancements in sensor technologies, certain parameters (e.g., *Escherichia coli*, total phosphorus, total nitrogen, and chemical oxygen demand, among others) still require the traditional laboratory approach for analysis due to a lack of suitable sensors [20,21]. This lack is due to the high sensor costs for these parameters, the need for the frequent cleaning process and calibration due to fouling issues, and the regular replacement of sensors due to their shorter lifetime [9,11].

Nonetheless, the algorithmic response of the propagation pattern and interdependency between WQ parameters can be an accessible and cost-effective alternative to these hardware sensors [22]. This software (or indirect approach) that processes the accessible secondary (or surrogate) data through models and enables prediction of the target parameter(s) is called a virtual (or soft) sensor [23]. An essential element in the performance of these virtual sensor systems is selecting the model to infer these values [24,25]. The three main approaches used to develop virtual sensing models include knowledge-based, mechanism-based, and data-derived (or ML) techniques [26,27,28]. The first two methods are relevant when the process mechanism is known or there is enough wealth of experience about the process [26]. However, these are often unavailable due to the dynamic nature of environmental factors and parameters that can influence WQ. 

Consequently, the ability of ML (a subfield of artificial intelligence) to extract useful information from an accessible historical database makes it ideal for virtual sensor applications [22,24,29]. In this context, the other significant contribution of the IoT-based WQM system is that it enables the integration of ML tools in the cloud server in order to predict these hard-to-measure parameters based on surrogates measured using sensors [16]. This essentially implies that monitoring costs can be significantly reduced since only parameters commonly measured in situ can be used as surrogates. The overall implication is that all critical parameters can be monitored in real-time, enabling early warning capability. 

Despite the reported success and advances of indirect measurement strategies in WQ assessment, especially in wastewater treatment plants [30,31], several shortcomings remain. For instance:

(i) There is limited information on surface and groundwater resources concerning a comprehensive overview of WQ parameters, particularly in the context of virtual sensing;
(ii) There is no clear specification book for an advanced water quality assessment (WQA) system;
(iii) In this study scenario (surface and groundwater), no study has reviewed very recent advances in ML concepts that have the potential to enrich the virtual sensing realization for WQA.

It could be observed that these constitute some of the critical challenges that should be addressed in order to promote the extensive use of virtual sensing in real-world applications. To the best of the author’s knowledge, there is currently no review in the literature that presents the current state of the art on virtual sensing systems for WQA of surface and groundwater resources. This work fills this gap and also addresses the limitations mentioned above by presenting a detailed analysis of the feasibility application of virtual sensing for online monitoring of surface and groundwater sources for a particular use case (or application). 

### 1.2. Work Objectives

This review seeks to achieve the following objectives:

1. To provide an overview of key WQ parameters for the particular water use. The overview will:
  1.1. Discuss the criteria for selecting WQ parameters and then identify (or provide) the key parameters that need to be monitored for the specified use case;
  1.2. Discuss the importance and traditional measurement process for each parameter;
  1.3. Provide the corresponding threshold concerning acceptable contamination;
  1.4. Provide the required accuracy for measuring each of the parameters;
  1.5. Formulate the measurement cost model (or estimate) for each parameter.
2. To discuss virtual sensing fundamentals (for dummies level);
3. To formulate a comprehensive specification book for an advanced WQA process (that involves a robust virtual sensing module) that has the potential to be an enabler for real-time (or near real-time) monitoring of WQ;
4. To identify and discuss the most recent advances in ML concepts that can enrich the virtual sensing realization for WQA.

The rest of the paper is organized as follows: firstly, an overview of key WQ parameters is given in Section 2. Subsequent to Section 2, fundamentals of virtual sensing are presented in Section 3. The specification book for an advanced WQA system is defined in Section 4 prior to recent advances in machine learning concepts potentially enriching virtual sensing realization for WQA being discussed in Section 5. Finally, the work is concluded in Section 6.

## 2. Water Quality Parameters: An Overview

### 2.1. Description of the Use Case

Generally, four broad categories of water use are known as water use for domestic, industrial, agricultural, and recreational purposes. These categories can be subdivided into several subcategories, such as water for cooking, drinking, manufacturing, livestock watering, irrigation, swimming, etc. Accordingly, the subcategories of specific uses have different WQ requirements. For instance, good quality river water that may be suitable for irrigation use may be unfit for cattle drinking or municipal use unless appropriate treatment for sediment removal is carried out [32]. Likewise, groundwater of good quality for municipal use or cattle drinking may be too corrosive for industrial use (e.g., boiler feed water) without suitable treatment for its corrosion potential [33]. An ideal situation would be to have several supplies from which to select water, but only one supply is usually available. Therefore, the quality of this available supply must be assessed for its fitness for the intended use.

For this reason, this work will focus on water use for agricultural purposes, particularly irrigation water. The choice is motivated mainly by the fact that agricultural water use is the most significant of all activities that require water since it accounts for around 72% of the total water consumption globally [34]. Since the 72% is even higher in arid and semi-arid regions like Southern Africa, the Middle East, and North Africa, the implication is that irrigated agriculture depends on an adequate water supply of acceptable (or suitable) quality [32,34].

### 2.2. Selection of Key Water Quality Parameters

The first step required in designing a WQA program is to establish the purpose of monitoring, as this would, amongst other factors, help select which parameters to monitor [6]. The decision of which parameters to choose for analysis depends, in the main, on the types of WQ problems and pollution sources believed or known to impact the receiving water body [35]. In this context, most countries concentrate on a few basic (or core) parameters, together with those specific to the planned water use or pollution condition. Basic parameters (e.g., pH, electrical conductivity, dissolved oxygen, etc.) are not direct measures of WQ for agricultural purposes and human or ecosystem health but are for characterizing the waterbody because deviation from normal ranges may be symptomatic of impacts on WQ [36]. Generally, users of irrigation water may experience a range of effects due to changes in WQ. The most common WQ-related problems in irrigated agriculture are those related to [32,37,38]:

- *Salinity*: salinity (known as the concentration of dissolved salts in soils and waters) problem exists when salt builds up in the crop root zone to concentrations that cause a loss in yield [32]. High salt concentration increases the soil solution’s osmotic pressure, a situation that can lead to physiological drought. That is, although the soil in the field may appear to have enough moisture, the crops will wilt since their roots will be unable to absorb the soil water [37,39];
- *Water infiltration rate*: the problem of infiltration occurs when the usual infiltration rate is significantly reduced to supply the crops with adequate water to sustain satisfactory yields. The two most common WQ factors impacting the normal infiltration rate include water’s salinity and its sodium content in relation to magnesium and calcium content [32];
- *Specific ion toxicity*: toxicity problem occurs when particular ions (or constituents) in the water or soil gets absorbed by the crops and accumulate to amounts high enough to damage the crops or reduced the yields. The main ions of concern include sodium, chloride, and boron [32,39]. Toxicity issues, which may occur even in low concentrations of these ions, often complements and complicates water infiltration or salinity problem [32];
- *Miscellaneous problems*: the other problems related to irrigation WQ include high nitrogen concentrations that may cause excessive vegetative growth; high concentrations of chemical oxygen demand that consumes dissolved oxygen and inhibits plant growth; and numerous abnormalities often linked with an unusual water pH [32,34]. Another significant problem faced by farmers using irrigation water is damage to irrigation equipment because of water-induced encrustation or corrosion [40].

In most cases, the national WQA program is designed to provide information on a large geographical area with several sampling points (or sites) [35]. For this reason, the selection of parameters to be measured is undertaken very carefully to provide maximum information at the minimum cost [6,7]. The parameters to be measured are chosen to ensure that the developed information will meet the requirements established in the first step of the design process. Considering the four common problems mentioned above, the general parameters for monitoring WQ for irrigation purposes, as identified by [32,37,38,39], are given in Table 1.

### 2.3. A Brief Discussion of Key Water Quality Parameters

The complexity of WQ as a subject is reflected in the numerous types of measurements of WQ indicators. For instance, some measurements (pH and temperature, for example) are most accurately made onsite because water exists in equilibrium with its surroundings. In situ (onsite) measurement techniques in the analysis of water samples require neither sampling nor sample preparation and therefore afford fast detection of substances at a minimum cost due to a reduction in the number of samples. This is important because careful (or proper) sampling and sample preparation are integral components of the whole laboratory value chain since they, in some instances, represent the main contribution to the error of the entire analytical process, even in cases where determinations are correctly performed [41,42]. The time between sampling and laboratory analysis may, at times, extend to hours or even days (especially in rural communities where regional analytical laboratories may be far), leading to chemical changes within the sample composition. Therefore, the traditional laboratory procedure for analysis is time-consuming, cost-intensive, prone to analytical errors, results in delays in data acquisition, and presents difficulties in detecting events that occur during the time interval between two samplings. Consequently, in situ techniques have been increasing in interest and application since they can overcome some of these drawbacks [41].

However, not all parameters can be economically (or easily) monitored in situ. For instance, *Escherichia coli* analysis is accurately made in well-equipped laboratories due to the complexity of such a measurement [43]. Therefore, although the analytical laboratory approach has several shortcomings, its comprehensiveness, accuracy, and adherence to standard techniques for regulatory and legal procedures have maintained its necessity [11]. While standardized testing techniques are available from several nationally recognized sources, we will in this review refer mainly to those given in “standard methods” [43] since this is a reference cited the most in water testing procedures (for instance, its twenty versions were cited 51,232 times as of 30 September 2021, based on Google Scholar). Below is a brief explanation of the importance and how each of the fifteen (15) parameters is traditionally measured.

#### 2.3.1. Potential of Hydrogen (pH)

pH is a measure of how acidic or basic (alkaline) the water is. Extremely low pH (acidic water) may accelerate irrigation system corrosion, while high pH (alkaline water) reduces the solubility of magnesium and calcium ions, leaving the sodium ion very dominant in the solution [34]. It is subject to change after sample collection, and, therefore, the measurement is traditionally made in situ using a pH probe [35,44].

#### 2.3.2. Electrical Conductivity (EC)

The EC, often simply called conductivity, is an expression of water’s ability to conduct an electrical current. As this property is related to the sample’s ionic content, it is useful for estimating water resources’ salinization [45], and therefore, one of the key parameters for determining water’s suitability for irrigation since high levels may deteriorate the water absorption of crops [34,37]. EC is a readily determinable parameter whose measurement is made in situ since it changes with storage time [44,46].

#### 2.3.3. Dissolved Oxygen (DO)

DO is the amount of non-compound (or free) oxygen available in the water. Its deficiency may lead to the lack of root oxygen, resulting in low crop growth and yield [34]. The most popular (or traditional) method for measuring DO is the in situ measurement using a DO probe [44,46].

#### 2.3.4. Chemical Oxygen Demand (COD)

COD refers to the oxygen amount consumed during the oxidation of all inorganic material and the breaking down of organic matter present in water. Elevated COD levels imply that a more significant amount of oxidizable organic material is present in the sample, which will ultimately reduce the DO levels. Traditionally, a sample of COD is refluxed in a strong acid medium with a known potassium dichromate excess. This traditional procedure takes about 2–4 h to complete [43,47].

#### 2.3.5. Total Nitrogen (TN)

Although nitrogen is critical for processes such as crops growth, leaf area expansion, and biomass yield-production, an overdose may result in excessive vegetative growth and delays in crop maturity [32]. TN compounds are analyzed by injecting the sample solution onto the in-line flow injection analysis (FIA) manifold [43]. Easy implementation is the primary advantage of FIA, whereas the high reagent and sample consumption are some of its main drawbacks [48].

#### 2.3.6. Sulphate

Sulphate in irrigation water (with appropriate amount) facilitates plant growth. However, its existence is a significant contributor to salinity [34]. The reference method is to determine dissolved sulphate turbidimetrically on precipitation as insoluble barium sulphate [43]. The method has a shortcoming that the results are not reproducible [49].

#### 2.3.7. Chloride

While chloride is essential to crops in small amounts, high concentrations can be toxic to sensitive crops [34]. It readily moves with the soil-water, gets absorbed by crops, and eventually builds up in the leaves, resulting in drying of leaf tissues or leaf burn [32]. One of the traditional methods for determining the chloride ion concentration is the Argentometric (or Mohr’s) method [43]. Although the procedure is simple and consumes less time, its drawback is that it is prone to errors due to the need for an excess titrant before the endpoint color is visible.

#### 2.3.8. Boron

Boron is an essential plant nutrient but becomes toxic to plant growth at higher concentrations [40]. Its toxicity symptoms are usually seen on older leaves as a yellow pigment or drying of the leaf tissues at the edges and tips [32]. The preferred method for boron analysis is the inductively coupled plasma method [43]. However, a major drawback of this method is its high capital cost [50].

#### 2.3.9. Sodium, Calcium, and Magnesium

Irrigation water containing high sodium is of particular concern because of its impact on the soil [39]. Sodium will change the soil characteristics and permeability (the ease with which liquids, gases, or plant roots pass through or penetrate a layer of soil or a bulk mass of soil), known as the “sodium hazard” [34]. This hazard is generally expressed as the sodium adsorption ratio (SAR). The SAR is determined from the ratio of sodium (Na^+^) to calcium (Ca^2+^) and magnesium (Mg^2+^) as [38]:

(1) $$\mathrm{SAR}=\frac{{\mathrm{Na}}^{+}}{\sqrt{\frac{1}{2}\left({\mathrm{Ca}}^{2+}+{\mathrm{Mg}}^{2+}\right)}},$$

where the SAR is expressed in meq/L. Calcium and magnesium are accurately analyzed with the atomic absorption spectrometric technique and the inductively coupled plasma method [43], while the most commonly applied method for sodium analysis is flame emission photometry [43,51]. However, laboratories in some developing countries do not have this modern equipment, and still rely on the somewhat error-prone titration method for calcium and magnesium and then determine sodium by calculation [37].

#### 2.3.10. Potassium

Together with nitrogen and phosphorus, potassium is a major plant nutrient. Typically, although potassium is found in smaller amounts in natural waters, high concentrations are usually found in recycled sewer water and can have adverse effects on water infiltration and the growth of plants [52]. Similar to sodium, the most commonly applied method for potassium analysis is flame emission photometry [43].

#### 2.3.11. Alkalinity

Alkalinity is primarily determined by the presence of carbonates, bicarbonates, and hydroxides in water [43,52]. It measures the capacity of water to neutralize acids [43]. These alkaline compounds (carbonates, bicarbonates, and hydroxides) eliminate the H^+^ ions in water (by combining with these ions to create new compounds) and lower their acidity (which translates to increased pH). Without this acid-neutralizing capability, adding any acid to a water source would immediately change the pH [52]. Alkalinity is traditionally determined in the laboratory by titration method that takes over 4 h to complete [43].

#### 2.3.12. *Escherichia coli* (*E. coli*)

*E. coli* is a fecal coliform bacterial species explicit to fecal material from warm-blooded animals (including humans) [43]. Its presence in water is a precise indication that many types of disease-causing pathogens may be present [53]. The standard for monitoring *E. coli* in irrigation water depends on the types of crops. Generally, edible crops that need cooking before ingestion have a low standard, while fruits or vegetables that can be digested directly have a high standard [20,34]. The culture-based laboratory method for estimating *E. coli* requires about 18 h of incubation to be cultured [20].

### 2.4. Irrigation Water Quality Indices

Comprehensive WQ analysis for its appropriateness for irrigation and evaluating the likely adverse effects of polluted water on soil and crop production is essential to mitigate contamination problems. However, WQ for irrigation purposes cannot be adequately determined by studying the factors affecting the irrigation WQ separately but rather by including several variables in a single numeric value [38]. This single normative value is called an irrigation water quality index (IWQI). This normative value, which represents the quality class of water, is an important tool used to understand the collective effects of various WQ parameters and enables the assessment of spatiotemporal changes to distinguish dangers to WQ for improved administration, evaluation, and utilization for irrigation purposes [38,54]. Several IWQIs have been developed, as shown in Table 2.

### 2.5. Regulatory Standards with Respect to Acceptable Contamination

Regulatory standards or recommendations of irrigation WQ are the norms commonly used as yardsticks to measure the effect of WQ on particular water use. Based on the IWQIs given in Table 2, the classification of surface WQ for irrigation purposes can be generalized, as shown in Table 3.

Although these IWQIs are used extensively for IWQ assessment, they still exclude some of the critical parameters, as shown in Table 4. The threshold concentrations at which different plants are affected vary over a considerable range. For instance, boron has a broad range from 0.4 (in Israel) up to 5 mg/L (in Egypt) [34,58]. The maximum COD allowed in Japan is 6 mg/L, while that of Italy is 100 mg/L [34]. While the detection of bacteria (fecal coliform and *E. coli*) is not allowed in countries such as the USA and South Korea, several other countries have a standard of up 10,000 cfu/100 mL [20,34]. Among the different WQ classifications reported in the scientific literature, the most used or internationally accepted is classification (or guidelines) reported by Ayers and Westcot [32].

### 2.6. Measurement Accuracy and Acceptable “Accuracy Tolerance” Ranges

All measurements have some degree of uncertainty attributable to either systematic or random errors [43]. Measurement accuracy is then defined as the difference between the output value of the sensor and the reference value (ground truth or some benchmark) of the output as measured by a perfect calibration standard. This measurement accuracy is vital because discussing the potential cost benefits of using sensors is usually relevant once the operational performance measures (e.g., precision, bias, etc.) for an application can be met [13]. Sensors and sensor systems are designed to give readings with predetermined accuracies, where the level of accuracy (and precision) will not be the same for each of the measured parameters. Among other factors, accuracy and precision depend on monitoring objectives [59]. 

For instance, as identified by the Environmental Protection Agency, each critical air pollutant must be measured within specific precision and bias (metrics for assessing the sensor’s accuracy) [60]. For regulatory monitoring, the metrics are specified as <7% for ozone, <10% for carbon monoxide, sulphur dioxide and particulate matter, and <15% for nitrogen dioxide [60]. These specifications are essential because precise and reliable measurements, whether through physical or virtual sensors, are required to ensure high-quality data are obtained to meet regulatory requirements. For water quality monitoring, particularly for domestic, aquatic, and agricultural use, accuracy benchmark data are often scattered and non-harmonized. This complicates information comparison across studies and, therefore, compromises transparency in the benchmarking and reporting of experimental case studies, especially data-derived prediction studies. For this reason, we will in this section collate measurement accuracies for each of the parameters (Table 1) as given in the literature to highlight and then propose the accuracy benchmarks for parameters whose accuracies are not given in the literature.

The accuracies, as given in Table 5, were extracted from several sources as described below. The first source is the mobile measuring stations (combines sensors and wet chemical analyzers) developed by Meyer et al. [61]. These measuring stations, designed as mobile trailers, are currently operated at 35 sites in Saarland (Germany), where they monitor 25 small and middle-scale rivers. The second source is a report that provides standard procedures and guidelines for use by US Geological Survey personnel to assess the Nation’s surface water quality [62]. The report provides guidelines for the site- and parameter-selection considerations, field procedures, data evaluation, sensor inspection and calibration methods, and data reporting. The third source is the design specifications from sensor manufacturers, such as Water Quality Sensors, Meters, and Measurement Systems —YSI [63], Aquaprobe-Aquaread [64], BOQU Instrument—Water Quality Sensor Supplier [65], Water Quality Monitoring—Xylem Analytics [66], and Online Water Quality Sensor—WINMORE [67]. The choice of these particular manufacturers is based mainly on the fact that they have publicly provided the accuracies for their sensors, and Rand Water (a water utility company in South Africa) currently uses a YSI multiparameter sonde for WQ analysis in the Vaal River system. A few other sensor manufactures have also provided their sensor accuracies. They are not reported here mainly because their accuracies are similar to those reported in these seven references.

TR, in Table 5, refers to proposed accuracy benchmarks. The rationale for these accuracies is that most of the sensors in practice, as seen from the references cited, including those not reported here, reach the accuracies within the proposed ranges and, therefore, a basis for those not specified.

### 2.7. Measurement Costs Models or Estimates

**Figure 1:**
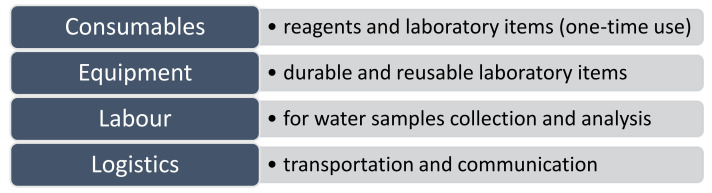
Cost categories for conducting a water quality test.

The increasing interest among researchers in developing low-cost WQ testing methods suggests that financial constraints are seen as the main barrier to testing [68,69]. Regardless, the economics of WQ analysis are not fully established, and therefore, the degree to which monitoring cost may be a limiting factor in different situations remains unclear. Generally, the cost of testing water quality can be classified into four categories [70], as shown in Figure 1.

Three studies [70,71,72] provided quantitative cost estimates for microbial drinking water quality monitoring. For cost data collection, Crocker and Bartram [72] relied on focus groups, interviews, laboratory observations, and sampling trips in seven developing and middle-income countries to estimate marginal costs (includes sample collection and transportation, consumables for testing the samples, and labour related to sample collection and analysis) of testing. In another study, Bain et al. [71] obtained the consumables costs from companies’ websites, catalogs, or quotations from suppliers for 44 microbial testing methods [71]. However, this study did not consider the labour cost and logistics, which is a significant drawback since a combined cost of labour and logistics constitutes about 75% of marginal costs [72]. Furthermore, both these studies relied on material cost data given by suppliers, manufacturers, and literature in certain instances and did not include importation taxes and transportation (or shipping) costs to laboratories after purchase. In a more recent study, Delaire et al. [70] utilized the actual cost data provided by eighteen monitoring institutions (ten health surveillance agencies and eight water utilities) in Sub-Saharan Africa to determine a microbial cost-per-test estimate. However, this study excluded logistical expenses and vulnerable (or unimproved) water sources (unprotected surface and groundwater) in the cost estimate.

Thus, the quantitative cost of a microbial WQ test or the precise cost calculation process thereof remains unclear. For instance, Delaire et al. [70] calculated the total monitoring costs by multiplying the cost per test and the number of tests for each person served with the population served. On the other hand, a technical report developed by the World Meteorological Organization and the United Nations Environment Programme proposes multiplying the number of sampling points with frequency and parameters to obtain a rough monitoring cost estimate [73]. For this reason, we will adopt a qualitative approach for estimating the cost of analyzing each of the parameters given in Table 1. We will first classify the parameter cost measurement into low, medium, high, or very high and then determine the overall cost estimate based on the current monitoring program. The classification will be based on:

1. *Sample preservation*: this will consider the required sample preservation and (or) recommended sample transportation time. Sampling and sample preservation may introduce serious errors due to failure to properly remove previous sample residues from sample containers, contamination from a sampling device, and loss of metals by precipitation and/or adsorption on sample containers caused by a failure to properly acidify the sample [43]. The COD analysis is one such case since its sample must be preserved by acidification to pH ≤ 2 using the concentrated sulphuric acid [43]. Noting the sample transportation time is essential, particularly for rural populations where the nearest regional laboratories can be miles away from source water supplies. For instance, the *E. coli* sample must preferably be analyzed within six hours of sample collection [71], and this may be impractical in such cases;
2. *Transportation cost*: the return of samples to central laboratories within a few hours depends, to some extent, on the availability of good road infrastructure and reliable motorized transport for sampling officers. Therefore, transportation costs (vehicle fuel and maintenance) will always be a factor whenever the samples need to be transported to the laboratory (lab);
3. *Labour*: this aspect will acknowledge the salaries for technicians involved in sample collection and testing. As pointed out by [72,73], sample collection, preservation, transportation, and lab analysis is the most resource- and labour-intensive phase in water quality monitoring;
4. *Equipment cost*: this will recognize costs of reusable (or durable) lab items such as refrigerators, culture tube racks, weighing scales, incubators, autoclaves, hot plates, magnetic stirrers, glassware, inoculation loops, etc. since some of the equipment rely on stable electricity supply and periodic maintenance or replacement;
5. *Consumables (quantity + safety)*: consumables include reagents costs and one-time use laboratory items like distilled water, absorbent pads, filter paper, alcohol disinfectant, gloves, cotton swabs, gas cylinders, etc. We will pay more attention to the quantity and safety of each reagent per parameter assessment. For instance, the COD test involves dangerous chemicals that need careful disposal (hazardous mercuric sulphate) and are potentially harmful (sulphuric acid) to operators [43];
6. *Duration of measurement*: this will assess the time it takes to complete the experimental analysis since the assessment time usually determines the feasibility of measuring the particular parameter in real-time [29]. For instance, sulphate determination takes less than 10 min, COD takes 2–4 h, while *E. coli* takes about 18 h;
7. *Communication + computing costs*: this will consider the costs associated with hardware and software for data storage, processing, interpretation, and reporting; production of outputs such as presentation software or geographic information systems. This step is critical since interpretation and reporting of monitoring results enable relevant stakeholders to make suitable recommendations for future actions [7].

Based on the measurement process for each parameter, the four cost categories are assigned numerical values as low (L): whenever the overall parameter cost estimate (cost) is ≤2; medium (M): whenever 2 < cost ≤ 4; high (H): whenever 4 < cost ≤ 6; and very high (VH): whenever cost > 6. Table 6 presents the final cost estimates. Each monitoring activity was scored as follows: an activity was given a total score of one (1) whenever the particular activity was entirely unavoidable (except for the duration of measurement). For instance, all in situ measurements have a score of zero (0) for sample preservation since samples are neither preserved nor need to be transported to laboratories within a particular time. On the other hand, total nitrogen, sulphate, and COD samples have to be preserved before transportation [43,74], although the transportation time is not critical (hence a score of 0.5). In contrast, the *E. coli* samples must be preserved and transported to the lab within six hours (hence a score of one). All the parameters analyzed in the lab have a transportation cost score of one since they must be transported to the lab for analysis. The same parameters have a score of one for labour, while those measured in situ have a score of 0.5 (for regular inspection of sensors and maintenance or repair performance when required). Equipment costs are zero for parameters measured in situ since we are not considering capital costs, while they are ones for all other parameters (except for chloride) since they all require a certain amount of lab equipment, some of which require regular maintenance or replacement. Chloride requires only a few apparatuses, hence a score of 0.5. Parameters that need many reagents were given a score of 0.5 and another 0.5 if some of those reagents or their corresponding wastes are unsafe. Duration of measurement was scored differently. An analysis that takes 0–30 min was given a score of zero; 30 min–1 h was scored 0.5; 1–12 h was assigned a score of 1, and an analysis that takes more than 12 h was allocated a score of 1.5. All the parameters were scored 1 for communication and computing costs since the data have to be stored, interpreted, and reported, regardless of whether it is measured in situ or in the lab.

As seen from Table 6, eleven of the fourteen parameters fall into the high and very high categories, and the average (or overall) cost estimate based on the current (or traditional) monitoring program is high since the average cost score is 4.5.

## 3. Fundamentals of Virtual Sensing

### 3.1. Physical Versus Virtual Sensors

**Figure 2:**
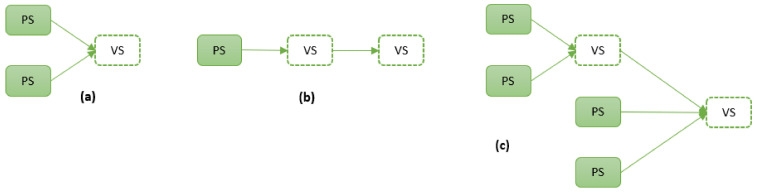
(**a**) a virtual sensor based entirely on physical sensors; (**b**) a virtual sensor based only on another virtual sensor; (**c**) a virtual sensor based on both virtual and physical sensors [75].

Generally, a sensor is defined as a device that measures a quantity based on the change in its environment [13]. A *physical sensor* reacts to a physical stimulus and then transmits the resulting impulse, usually by means of electrical signals storable in digital form [75]. For example, some temperature sensors consist of a thermistor whose electrical resistance changes due to a change in temperature [76]. During the device’s calibration, a scientist would determine a formula that converts the ohmmeter (an electrical instrument that measures electrical resistance) readings into temperature [77]. Although this conversion formula is a simple linear relationship, it is important to note that even common sensors rely on a model (or formula) to convert the effect of note (resistance in this case) to that being measured (temperature in this case). Contrary to physical sensors, a *virtual (or soft) sensor* is entirely a software sensor that autonomously produces signals by aggregating and combining signals that it receives from physical or other virtual sensors (whether synchronously or asynchronously) [75,78]. Figure 2 demonstrates three virtual sensor (VS) constellations: (a) a vs. based entirely on physical sensors (PS), (b) a vs. based only on another VS, (c) a vs. based on both virtual and physical sensors [75,79].

As seen from these constellations, virtual sensing relies on data captured by physical sensors. The data delivered by physical sensors are embedded into software applications that execute algorithmic analytics on these combined data sets. This fusing and processing of several sensor inputs enable VSs to measure process parameters that may not be physically measurable themselves [80].

### 3.2. An Introduction to Virtual Sensing (for Dummies Level)

In simpler terms, a data-derived vs. can be defined as a formula (or inferential model) that converts several inputs (easy-to-measure secondary parameters) from cheaper sensors and combines them to infer the outputs (hard-to-measure primary parameters) of the more complex or expensive sensors [30,81]. As an example, let us discuss the commercial sensor for COD measurement. Directly measuring COD is possible, but the sensor is prohibitively expensive (ranges from $1500 to $3000) [22], especially for long-term in situ mass deployment and bound to experimental error (particularly for sensors that use total organic carbon as chemical oxidants) due to the partial (instead of full) oxidation of some organic and inorganic matter [82]. With the possibility of replacing this sensor with a formula that depends explicitly on the much cheaper (but robust) sensors (e.g., pH, temperature, DO, flowrate, conductivity, etc.) [22,83,84], then we would have several benefits from this. For instance [77]:

1. The vs. value is cheaper both initially and in the long run since no equipment needs to be bought or maintained;
2. It is ideal for real-time monitoring since the vs. will never be removed for issues such as recalibration;
3. It is also ideal for high-frequency monitoring since there is no need to wait for a long chemical reaction to take place;
4. It can be easily scaled over many locations without extra investment.

Therefore, replacing these expensive sensors with VSs makes sense since VSs can, to some extent, eliminate the inefficient laboratory process and enable the real-time (or near real-time) monitoring of parameters that are hard-to-measure. The idea of a vs. is similar to that of the thermistor since we are measuring a basic quantity like electrical resistance that is eventually converted into what we ideally wanted to measure (here, the temperature). The main differences are that we now have several basic parameters flowing into the computation and, generally, a nonlinear relationship among these parameters to obtain the required output [77]. These present a dual challenge.

*First*, enough domain or system knowledge must be collected in order to answer the fundamental question: which basic measurements are essential for computing the parameter of interest? Leaving out critical parameters may result in a poor model, while including parameters not connected to the output may decrease the inference performance [24]. For dummies, let us use an everyday example to explain this. There is a correlation between the number of swimming pools drownings and ice cream sales. While this correlation (an occurrence (or action) that directly links to another occurrence, although this link does not automatically imply that the change in one is the cause of the change in the other) is true, this relationship is clearly not causal. Higher ambient temperature is clearly the main driver of the increase in ice cream sales including more pools visits, which ultimately increases the drownings. Therefore, ambient temperature and not the number of drownings should ideally be used to predict ice cream sales. This is certainly logical to human beings (domain experts) who know what is going on. However, this is not as obvious to computer analysis techniques that only look at correlations. *Second*, the relationship between all the parameters must be established in order to find the best model to compute the desired output. This is the core of machine learning (discussed in Section 5).

### 3.3. Virtual Sensor Development

**Figure 3:**
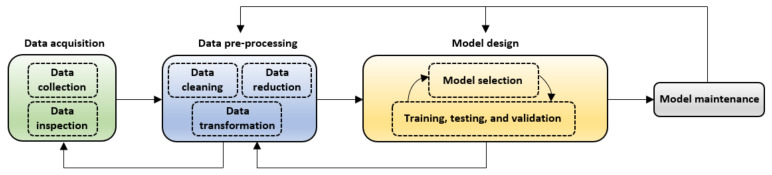
An overview of the typical steps undertaken in developing data-derived virtual sensors [25,30].

This section discusses the primary steps considered in the development of VSs. An overview is presented in Figure 3, and the commonly used methods are discussed thereafter. This procedure (which is an iterative process) is not explicitly standardized, although widely adopted by both practitioners and researchers [25,30,79,85,86].

#### 3.3.1. Data Acquisition

Improving WQ is an essential part of the United Nations sustainable development goals, and data collection and sharing are crucial steps for reaching the associated WQ targets [87]. *Data collection* is the first step in designing (or developing) data-derived VSs, and therefore access to credible water quality data is critical for developing (training, testing, and validating) these models since low-quality data will lead to low-quality models (sensor data quality is generally a universal requirement [13]). For this reason, some studies utilize publicly available data from well-established regional databases such as the water quality portal [88] and the center for ecology and hydrology [85]. Data collection is then followed by *data inspection*, which is performed to overview the prominent data structures and identify any obvious problems [25,30]. As viewed from the vs. development and maintenance point of view, the most common characteristics (or problems) of the process industry data include sampling rates and measurement delays, missing values, data outliers, data drifting, and data co-linearity [25]. Due to these characteristics, the pre-processing of data remains critical for vs. development.

#### 3.3.2. Data Pre-Processing

This step transforms the experimental data in a way that they can be processed more efficiently. The typical pre-processing steps include data cleaning, transformation, and reduction. *Data cleaning* routines attempt to “clean” the data by handling the missing values, dealing with the noisy data, and resolving data inconsistencies [89]. *Data transformation* is undertaken to transform or consolidate the data into appropriate forms suitable for the data mining process. It involves strategies such as normalization, variable (or attribute) selection, discretization, and concept hierarchy generation [90]. *Data reduction* techniques are utilized to obtain a reduced data representation that is relatively smaller in volume while still producing analytical results that are the same or almost the same. Data reduction strategies include variable subset selection, data compression, numerosity reduction, and dimensionality reduction [89].

The goal of variable subset selection is to reduce the data size by eliminating redundant or irrelevant inputs such that the resulting probability distribution remains as close as possible to the distribution obtained using all original attributes [89]. This step is fundamental because the premise for vs. deployment is to utilize the minimum number of physical sensors to minimize these sensors’ capital and maintenance costs since they directly feed the vs. [85,91]. Therefore, the two prevalent limitations of virtual sensing are that (i) it relies on data captured by physical sensors (this may present physical or economic limitations) and (ii) relies on the use of appropriate models to combine them in order to estimate (or infer) the variables that are difficult or expensive-to-measure.

#### 3.3.3. Model Design

This step is very critical in vs. development [25,30], for the model structure defines the specific use case and documents the developer’s assumptions on the problem being solved. Furthermore, selecting an optimal model type is vital for the vs. performance and also determines the sensor’s generalization ability. However, there is currently no standard approach for performing this task, and therefore, the model’s topology and its hyperparameters are usually chosen in an ad hoc fashion for each vs. [25,30]. This is because model design (or selection) is task-dependent and is often based on the developer’s personal preference and experience. This is observable in published vs. applications, especially in water quality assessment, where many designers focus on a single model type (e.g., artificial neural networks) in their field of knowledge. 

Nonetheless, despite the unavailability of a theoretically established approach to model selection (or design), two main tasks are prevalent:

(i) Model structure selection;
(ii) Model training, testing, and validation.

The normal practice is to start with a simple model type, evaluate its performance, and then gradually increase the model complexity, provided that there is substantial improvement in the model’s performance [25,30,92]. Moreover, it is essential that the developed models are accurate, interpretable, and computationally efficient to promote extensive use of virtual sensing in practical applications [24,30,85].

#### 3.3.4. Model Maintenance

After successful development and deployment, the vs. has to be maintained and updated regularly. This maintenance is essential because of drifts and other data changes, which results in deteriorating vs. performance which must be compensated for by either adjusting or re-developing the model [25]. However, their manual redesign must be avoided owing to the heavy workload during feature engineering [30].

### 3.4. Current Status of Virtual Sensor Applications for Water Quality Assessment

This study focuses primarily on the application of virtual sensing for surface and groundwater quality assessment since the most common sources of irrigation water include surface and groundwater [93]. Surface water sources include “flowing” water supplies (i.e., rivers, canals) and “standing” or stored water supplies (i.e., reservoirs, lakes), while groundwater supplies may come from springs and wells. To identify relevant papers, we searched for articles published in the past 20 years (2001–2021) associated with vs. applications for surface and groundwater quality assessment. It is expected that such a time period is adequate for the overall state-of-the-art progress in this study domain. The papers were identified as follows. Firstly, “virtual sensor” OR “soft sensor” AND “river” AND “water quality” (excluding wastewater) were the keywords used to search for papers in Google Scholar. The part “river” was then interchanged for other sources such as “lake,” “reservoir,” or “groundwater.” Secondly, the abstracts and conclusions of these papers were reviewed to identify the relevant articles.

Furthermore, the above keywords were used to search across several influential water-related journals such as Water Research, Water Resources Research, Water Quality Research Journal, and Water Science and Technology. Finally, the list of references of the selected research articles was investigated to identify further references, and the process was repeated until the citation trail stopped. A review of these papers was then undertaken in terms of the modeling approaches used, input and output parameters, data collection time scale and sampling frequency, and whether the input data were pre-processed. A detailed description and analysis of the sixteen relevant papers are shown in Table 7.

#### 3.4.1. Commonly Used Modeling Approaches

**Figure 4:**
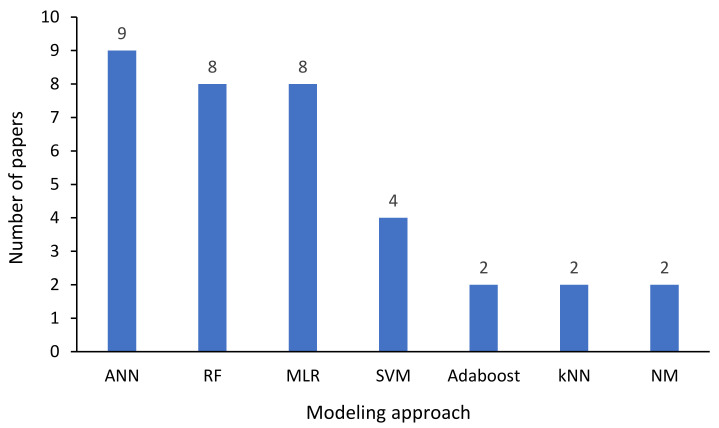
Techniques that were used the most in the papers reviewed.

The application of virtual (or soft) sensing for surface and groundwater quality assessment is emerging. This is evidenced by the low number of publications (16) and the fact that 63% of the articles [22,29,55,85,94,95,96,97,98,99] were published in the last three years (2019–2021). Therefore, as seen in Figure 4, it is not surprising that artificial neural network (ANN) related algorithms are the most applied ML techniques.

This popularity agrees with the observation of other reviews on the application of ML for WQA [88,106,107]. Apart from their more accessible calibration, robustness, and capability to process nonlinear and complex datasets [106,107], this trend can be attributed to the ANN technique requiring a fairly small amount of data to produce satisfactory prediction results [108]. Even though random forest (RF) and multiple linear regression (MLR) were both predicted eight times, the high number of RF applications can be attributed to the fact that RF is classified as follows [85,109,110]:

(i) The simplest compared to other ML algorithms;
(ii) One of the most successful ML techniques in practice (particularly from transferability to the end-user point of view) since the models are not very sensitive to noise and outliers.

In contrast, the high number of MLR applications can be somewhat attributed to most studies investigating the feasibility of utilizing turbidity as a single surrogate to predict suspended solids (SS) and total phosphorus (TP) (discussed in Section 3.4.2). Generally, TP, particularly in rural settings, can be correlated with the concentration of SS, which itself is highly related to turbidity [85,105,111]. Although support vector machine (SVM) has only been applied in four different studies, it can produce excellent accuracy compared to MLR and ANNs [106]. Adaptive boosting (Adaboost), k-nearest neighbour (kNN), and numerical models (NM) were all applied on two occasions, opening up the scope for more analysis to test the effectiveness of VSs based on these techniques.

It should be noted that Figure 4 only presents the techniques that were applied on more than one occasion. Generally, two studies (excluding those that applied RF) used hybrid models [29,98], which were, on both occasions, found to outperform their single model counterparts. Hybrid models are developed by integrating different ML methods with optimization methods to advance the technique in various aspects [112]. For this reason, these models exploit the strengths of the combined methods to reach better performance compared with single models. The use of more hybrid models in this problem domain remains open for further research since only three (RF, support vector machine-sequential minimal optimization, and hierarchical clustering and k-means algorithm-fuzzy neural network) have been applied in the papers reviewed. Deep learning techniques are also yet to be tested as possible machine learning-based VSs. A brief discussion of some of these techniques is given in Section 5.

#### 3.4.2. Water Quality Parameters Modeled

**Figure 5:**
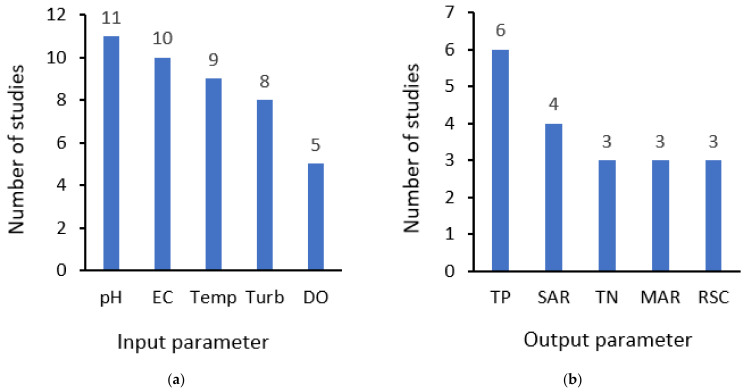
(**a**) Commonly used input parameters; (**b**) Commonly predicted parameters.

The premise of the data-driven virtual sensing technique is to use easily measurable parameters (or surrogates) as inputs to construct prediction models with hard-to-measure parameters as outputs. Thus, this section discusses the parameters commonly used (applied more than two times) as inputs (Figure 5a) and those used as outputs (Figure 5b).

Figure 5a indicates that pH is the most frequently used input parameter (in 11 of the 16 articles considered), followed by EC (10 papers), water temperature (temp) (9 papers), turbidity (turb) (8 papers), and DO (5 articles). This can be attributed to the fact that the sensors for these WQ parameters are relatively cheap [14], hence appropriate surrogates for hard-to-measure parameters since they can be easily monitored with a high time resolution. Although this is considered good practice, there are isolated incidents where parameters classified as hard to measure, based on Table 6, were used as inputs. For instance, Ha et al. [96] used COD as one of the inputs in predicting total phosphorus and total nitrogen.

Figure 5b presents the parameters that were predicted the most. As seen from this figure, total phosphorus (TP) was the most frequently predicted parameter. This can be partially explained by the fact that:

(i) TP concentrations change very rapidly with discharge, and the traditional grab sampling method is usually insufficient to capture the variabilities of TP concentration patterns [101];
(ii) Despite the interest in measuring TP, the sensor technology for continuous measurement of TP concentrations surface waters has not been developed yet [101];
(iii) TP is one of the nutrients whose presence in excessive amounts in water bodies leads to eutrophication; a condition that has caused a series of WQ problems for freshwater and marine ecosystems around the world [113].

TP prediction was followed by sodium adsorption ratio (SAR). As mentioned previously, SAR is a crucial irrigation WQ parameter used to manage sodium-affected soils since it (together with salinity) quantifies the infiltration problem. Generally, the infiltration rate decreases with either increasing SAR or decreasing salinity, implying that the two factors must be considered together to properly assess the ultimate impact on water infiltration rate [32]. Although TN is another nutrient whose excessive amount leads to eutrophication, the lower number of studies (4) compared to TP (6) can be explained by the fact that TP pollution is generally a more significant issue than TN [114]. The other two irrigation WQ parameters that were regularly predicted are residual sodium carbonate (RSC) and magnesium adsorption ratio (MAR). The interest in their prediction (or monitoring) can be explained by the fact that RSC indicates the hazardous effect of alkalinity on irrigation WQ [38], while MAR above 50 causes harmful effects on the soil [115].

Interestingly, of the parameters whose monitoring cost was categorized as very high, COD was predicted on only one occasion [22], while *E. coli* prediction was never studied. Since COD levels determine the amount of root oxygen available (in the form of DO), the lack of COD prediction studies could be mainly because the application of virtual sensing for irrigation WQ assessment is relatively new. For instance, only four studies in the papers reviewed explicitly stated irrigation purposes as the use case [55,95,99,102]. On the other hand, the reason for the lack of *E. coli* prediction studies is because the focus is mainly on the physical and chemical characteristics when determining the suitability of water for crop production [116]. However, the analysis of the biological characteristic of water becomes essential when sewage water is being used for crop production. For instance, the European Parliament approved a regulation (in 2020) for reusing treated wastewater for agricultural purposes where *E. coli* concentration limits range between 10 and 100 cfu/100 mL based on the irrigation techniques and type of crops [20].

#### 3.4.3. Data Collection Time Scale and Sampling Frequency

The accuracy and reliability of sensors (physical or virtual) are of primary concern when analyzing and monitoring water quality [11]. Amongst other factors, the availability of suitable, high-quality data plays a crucial role in determining how accurate and robust a developed inferential model is since data-driven approaches depend on good training data [117]. In this section, data quality in terms of collection time scale and the sampling frequency is discussed.

##### Data Collection Time Scale

Consideration of seasonal effects in WQ assessment is vital since WQ variables are often subjected to high variation in concentration based on the time of the year. Based on the papers reviewed, it is observed that four studies [22,95,97,100] did not report on the data collection interval used in building their predictive models. This points to the need for a more rigorous scientific reporting of data collection in data-driven modeling papers. Of those reported, two used data ranging from three [29] to four months [103]. This raises concerns about the robustness and reliability of the models developed since the input data does not cover all possible seasonal variations. Furthermore, these models could be prone to over-fitting (a situation where the model is very accurate on the training data but performs unsatisfactorily on previously unseen test data) due to fewer samples, casting doubts on the generalization ability of the developed models. Two other studies [94,102] utilized datasets spanning at least a one-year period, which may be considered sufficient enough to reveal the annual cyclic pattern of water pollution levels. However, there may be cases wherein the said period may not be adequate, especially in drought-stricken environments where extreme water pollution levels may not be represented well enough. Except [93], the remaining studies considered a satisfactory period covering all possible seasonal variations, which is generally considered a good practice.

##### Sampling Frequency

Continuous high-frequency WQ monitoring has become a critical task to support water management initiatives [85]. However, the sampling frequency for many WQ monitoring programs is generally very low (i.e., monthly) to accurately estimate nutrient loadings [94]. Based on the current review, nine studies (56%) did not disclose the frequency at which the data was sampled, while other studies used data that were sampled either monthly [98,101] or bi-monthly [96]. The rest of the studies (25%) used data that were sampled hourly or daily. Considering that the data sampling frequency significantly affects the performance of WQ modeling [88], the lack of transparency in the sampling frequency may cast doubts on the optimality of the reported results. Using monthly or bi-monthly data collection may be inappropriate, especially in pollution-stricken environments that occasionally undergo sporadic acidic episodes, nitrate peaks, or algal blooming [118]. Therefore, frequent sampling may be necessary during rainy seasons, low-flow conditions, or other regular industrial and agricultural activities. In essence, the need for alternative monitoring technologies that are quicker, effective, and inexpensive remains.

#### 3.4.4. Data Pre-Processing

Surface and groundwater studies usually use many parameters related to the properties of the water body, which can be redundant or highly correlated [119]. Thus, it is always advisable in any machine learning model to select a subset of relevant predictors (or features) based on the target pollutant [85]. This feature selection process increases the accuracy of the models by alleviating the curse of dimensionality (caused by a large number of input parameters and a limited number of samples [81]) and enhances generalization capability due to overfitting avoidance [119]. Additionally, a less complex predictive model with fewer features will require minimal resources to train and, therefore, will increase the model interpretability by the end-user [85]. Based on Table 7, two studies [97,100] did not give any detail concerning the data used, and therefore it is not clear whether it was processed or not. Seven studies pre-processed the data from the fourteen that provided some details while the other seven did not. Of those that did not, the number of predictors in certain instances was too large. For example, Harrison et al. [94] used 8 parameters, Sepahvand et al. [99] used 11 parameters, while Wagh et al. [102] used 13 parameters. Besides the effect on accuracy, complexity, and transferability of the models, using this large number of predictors defeats the purpose of virtual sensing if the aim is to reduce operational and maintenance costs on physical sensors. 

### 3.5. An Update of the Measurement Cost Estimate

The cost benefits of a low-cost monitoring program will be fully appreciated if the overall cost-benefit of such a system is clearly contrasted with the current monitoring program. Based on Figure 5b, parameters that have been successfully predicted in a virtual sensing mode include TP, TN, SAR (Na^+^, Ca^2+^, and Mg^2+^), MAR (Ca^2+^ and Mg^2+^), and RSC (alkalinity, Ca^2+^, and Mg^2+^). Although COD was only predicted once while *E. coli* was not predicted at all; it is well documented that they are virtual sensing parameters due to their long response time and high sensor cost [20,22,120]. Bearing this in mind, the updated cost model compared to that given in Section 2.7 (Table 6) is shown in Table 8.

As seen from Table 8, the overall cost estimate drops from high (4.5) to medium (2.3). This is huge, particularly for farmers in developing countries, who may not have sufficient resources for irrigation WQ monitoring. Considering that potassium (which is included in the computation of exchangeable sodium percentage) was predicted on two occasions [55,95], updating its total score to one brings the overall monitoring cost estimate to two, which is categorized as low (refer to Section 2.7). In essence, VSs can play a central role in developing innovative WQ monitoring systems. Furthermore, the studies’ results demonstrated that ML models coupled with automated sensor technologies could improve WQ monitoring. 

## 4. A Specification Book

Continuous high-frequency WQ monitoring has become a critical task to support water management [85]. Analyzing WQ requires understanding, modeling, and real-time monitoring of water pollution using automated WQ sensors through an IoT framework [29]. In this section, we formulate a specification book for an advanced WQ assessment system (that involves a robust virtual sensing module), which can be an enabler for real-time (or real-time) monitoring of WQ in surface water resources. Even though irrigation water supply depends mainly on the surface and groundwater resources, we will formulate the specification book only for surface water resources. The following assessment measures are the most relevant ones for a realistic real-world implementation scenario:

### 4.1. Parameters That Must Be Measured (or Observed) Continuously

Although ML models demonstrate acceptable accuracy in predicting and evaluating WQ, the monitoring efficiency also depends on the type and the number of input parameters (or predictors) used [55]. This implies that increasing the number of predictors analyzed in the lab decreases the efficiency of these monitoring systems and their field application. Furthermore, the IoT-based WQ monitoring setup requires real-time sensing [29]. Therefore, the requirement is to use a minimum number of physical sensors that directly feed the VS. For this reason, parameters that must be measured continuously are dependent on the cost of their sensors. Thus, pH, EC, DO, turbidity, and water temperature are parameters with low-cost sensors and can be measured in real-time, as in the studies reviewed (see Figure 5a). Therefore, it is proposed that these become the basic parameters to be considered surrogates for irrigation WQ monitoring. The significance of these parameters is that they are not only crucial for irrigation WQ assessment but are among the basic (or core) parameters that should form the foundation of almost any surface WQ monitoring program [121,122].

Although these parameters are commonly measured in situ, including all five may not be worthy in terms of improving the predictive accuracy (based on the monitoring objectives), as demonstrated in [85]. Therefore, for selecting the optimal independent parameters, it is important to note that deep learning models (briefly discussed in Section 5), such as the variable-wise weighted stacked autoencoder and advanced versions of generative adversarial networks, do, amongst others, automatically learn and integrate the dependency amongst the input parameters of the model(s) [123]. This is critical since it enables the utilization of a minimum number of parameters without compromising the predictive accuracy.

### 4.2. Specifications for Input and Output Parameters

For each of the input and output parameters, we define the following:

#### 4.2.1. The Respective (or Recommended) Accuracy Tolerance Ranges for Predictors

The two prevalent research themes in irrigation water management include water quality and quantity. Water quality, which is the primary focus of this study, is monitored mainly to control salinity and infiltration problems [32]. On the other hand, the growing water scarcity and the increasing competition for freshwater resources have resulted in stricter regulation of water used for agriculture [124,125]. This necessitated the development of more efficient irrigation practices without compromising crop quality and yield [125]. One of the most employed strategies for improving irrigation water use efficiency is through irrigation scheduling, i.e., using an adequate amount of water at the right time [126]. Recent developments in sensor technologies have enabled automated irrigation scheduling using soil moisture sensing devices [124,126]. Soulis et al. [126] investigated the impact of soil moisture sensors’ accuracy and positioning on irrigation scheduling systems. It was found that ±1% sensor accuracy affected the irrigation efficiency by 2.5% to 6.4%, while ±3% sensor error affected the efficiency by 10.2% to 18.7%. These results demonstrate the importance of accurate and reliable sensors. Therefore, in the absence of specified accuracy tolerance ranges for the selected WQ parameters, we will infer the recommended ranges from [126]. Consequently, all these parameters must be measured within the target accuracies, as shown in Table 9.

#### 4.2.2. The Recommended Accuracy Tolerance Ranges for Predicted Parameters

Although VSs estimate the values of objective parameters with a high degree of accuracy, their use has a few practical difficulties. One of the main difficulties is the degradation of vs. models [127]. The predictive accuracy of VSs decreases gradually due to factors such as strong process nonlinearity, predictors sensor drift, inappropriate selection of the input parameters, insufficient number of samples for model construction, inappropriate selection of the samples for model construction, among others [128]. Considering the implication of sensors accuracies based on [126], we recommend that the accuracy tolerance ranges for predicted parameters be within the acceptable limits as given in Table 5.

#### 4.2.3. The Realistic Measurement Frequency

Generally, some irrigation waters can damage the soil structure, while others damage crops directly [129]. The impact of irrigation water on plants and soil depends on the water, crop, soil, and environmental conditions. Therefore, testing (or measurement) frequency is based on water use and source. Nonetheless, surface waters are subject to flow patterns due to seasonal variations and may need frequent monitoring [129]. Since the four studies [55,95,99,102] that investigated WQ monitoring for irrigation purposes did not provide the measurement frequencies for the data utilized, the ideal practice would be to consult farmers or experts in the field. In the absence of this expert recommendation, we propose a year-round instream sonde deployment, especially in locations with warmer water temperatures whose sites do not freeze during winter. Data collection intervals can range from 1 to 10 min, and the data quality can be reviewed monthly via visual inspection of time-series plots.

### 4.3. The Global System Architecture of Virtual Sensor Monitoring in an IoT Environment

**Figure 6:**
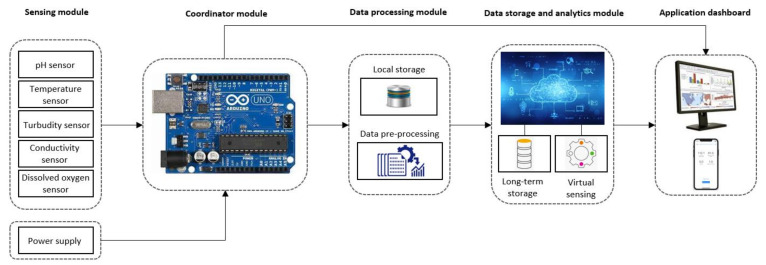
A virtual sensing architecture in an IoT environment [15,131].

Despite the alarming rate at which surface water quality is declining, the problem remains unaddressed to a large extent, particularly in developing countries. Several research efforts have been made regarding the application of the internet of things (IoT) for real-time WQ monitoring. However, the proposed IoT architectures do not consider the virtual sensing aspect [15,130,131] or include laboratory measurements as part of the input data [22,29]. Consequently, this section describes the global system architecture that involves virtual sensing and can build the basis for real-time (or near real-time) monitoring of WQ. The high-level architecture comprises several modules, including the sensing module, the coordinator module, the data processing module, and the data storage and analytics module, as shown in Figure 6. A detailed description of each of the modules is given next.

#### 4.3.1. Sensing Module

The sensing module contains inexpensive pH, temperature, turbidity, dissolved oxygen, and electrical conductivity sensors. These sensors, placed in the water to be monitored, convert the water quality parameters into equivalent measurable electrical quantity, which is transmitted to the coordinator module.

#### 4.3.2. Coordinator Module

The coordinator module links the sensing and data processing modules. The module usually uses the Arduino microcontroller to receive all the parameter measurements from the various in situ sensors connected to it [16,130]. The system includes a cellular modem and a data logger that allows the transmission of this data to a dedicated website or control room equipped with an alert facility to inform the user of any alarming deviation of water quality parameters.

#### 4.3.3. Data Processing Module

This module, which is connected to the coordinator module through a transceiver, includes web services such as local storage and data pre-processing services. Once the real-time measurements are received from the coordinator module, they are stored in a local database using the storage service. Additionally, the data pre-processing service processes the received data in real-time, including the filtering of useful data [15].

#### 4.3.4. Data Storage and Analytics Module

This module accomplishes two significant responsibilities. Firstly, to ensure the long-term storage of WQ data and secondly, to predict WQ using ML techniques [15]. Once the data have passed the pre-processing stage, it is then transferred to the cloud, where ML algorithms are applied for predictions. In the cloud, surrogate measurements can then be used to predict irrigation WQ parameters (or indices) that are expensive (or hard) to measure. The predicted parameters (or indices) can then be used to classify irrigation WQ based on the guidelines given in Table 3 and Table 4 or pre-defined acceptable ranges.

#### 4.3.5. Application Dashboard

The application dashboard, which is synchronized with the cloud to get real-time data, is used to visualize the WQ data (in the form of graphs and heat maps) on the web, mobile, and desktop platforms. The acceptable ranges can also be displayed on the interface and alerts (early warning) notifying the operators regarding any parameter(s) that seems to be gravely out of limit.

### 4.4. The Updated Cost Model: The Global System Architecture Included

The Global System Architecture (Figure 6) comprises hardware and software solutions. The hardware solution (located onsite) consists of a microcontroller, water quality measuring sensors (or multi-parameter probe), and a Wi-Fi shield for sending data to the cloud and the application dashboard. The software solution (in the office or control room) consists of a computer or mobile application. All these components (including the microcontroller housing and the structure that holds the immersed sensors) will affect the capital and operational cost. However, similar costs would still be incurred in the laboratory, although they were not considered in our cost model. For this reason, updating Table 8 by including temperature and turbidity under in situ measurement and assigning a score of 1 (for communication and computing costs) for all other parameters results in a total score of 1.2, which is classified as low. In comparison, the cost model described in Section 2.7 was high (4.5) while that in Section 3.5 was medium (2.3). Cutting the cost from 4.5 to 1.2 demonstrates that the solution to effective WQ monitoring lies in the integration of IoT in the monitoring system. The effectiveness of machine learning as a predictive tool was established. Therefore, researchers need to start developing prototypes where these systems are tested in practical settings to harness the full potential of the latest advancements in monitoring technologies. In the reviewed literature, only three articles validated their proposed approaches in a practical environment [22,29,98]. This implies that there is still a gap between academic motivations (theoretical interests) and the needs of those responsible for WQ monitoring. 

## 5. Recent Advances in Machine Learning Concepts

Generally, a wide variety of ML and statistical inference techniques have been employed to develop data-driven VSs, among which representative examples include artificial neural network (ANN), principal component regression that incorporates a regression model with principal component analysis, support vector machine (SVM), random forest, and partial least squares regression, where ANN and SVM are the most utilized [132]. Despite the promise of VSs, open (or unsolved) problems, especially those arising in the design of these sensors, have thus far limited the extensive utilization of this data-driven approach [133]. Some of the challenges arising in industrial (or practical) applications include [133,134]:

(i) Labelled data scarcity;
(ii) Data quality (outliers, missing values, measurement noise, etc.);
(iii) Unsupervised feature exploitation;
(iv) Computational complexity reduction;
(v) Virtual sensor maintenance.

Besides, one of the common problems of neural network-based VSs is their inefficiency to represent highly complex nonlinear phenomena, as a result of limited experimental data [133], a common problem in developing countries due to the lack of established WQ monitoring programs [135]. Although more complex representations are obtainable using deeper structures, it is difficult to train them using the traditional back propagation-like algorithms due to exploding and vanishing gradients [133]. On the other hand, SVM enjoys the advantages of accessible optima and low computational costs. Additionally, it effectively tackles the small data problems and has subsequently been used extensively as a vs. [136,137]. However, its drawback is that computational complexity grows with the increasing number of training samples [132]. Despite some reported successful applications, SVMs and shallow NNs do not provide an allowance for latent variable subspace, resulting in poor interpretation capability [132].

Recently, deep learning (a subset of ML that achieves great flexibility and power by learning the world’s representation as a nested hierarchy of concepts) emerged as an appropriate approach for solving some of the challenges mentioned above [26,133]. Due to deep learning (DL) techniques, computational models developed from several processing layers can learn complex data representations with various levels of abstraction [26,27]. By far, these methods have attained state-of-the-art breakthroughs in fields such as speech recognition, image and natural language processing [138]. Surprisingly, the first paper to develop a data-driven vs. based on DL only appeared in 2014 [133]. The paper uses a DL technique to design a vs. to estimate a crude-oil distillation unit’s heavy diesel cut point [132]. The advantages of DL over traditional methods are widely discussed in the paper. For instance, DL:

(i) Improves the representation ability;
(ii) Is efficient in handling massive data;
(iii) Enables the extraction of nonlinear latent variables;
(iv) Offers the possibility of utilizing unlabeled data.

Since 2014, several studies, particularly in industrial processes, have applied DL for predicting process variables that are hard-to-measure [133,139,140,141,142,143,144,145]. A detailed discussion of the current state of the art of virtual sensors based on DL is given in [133]. However, this section briefly describes some of these DL techniques that can enrich the virtual sensing realization for WQ assessment. The discussion will illustrate the potentialities of each proposed approach in virtual sensor design in response to the open problems mentioned above since these issues would have to be addressed in order to effectively apply DL for WQ assessment. From the discussed DL techniques, selecting the one optimal for a particular monitoring program remains a critical exercise. Szeląg et al. [146] recently proposed a virtual sensor expert system concept for selecting the optimal method. Amongst other aspects, the method considers the:

(i) Complexity of the predictive method;
(ii) Analysis time of input parameters;
(iii) Reliability of the measured data.

### 5.1. Auto-Encoders (AEs)

An AE is an unsupervised NN used to automatically learn features from unlabeled data (i.e., by reproducing its input at the output layer) [147]. To accomplish this, the AE independently learns how to compress data from the input layer into a shorter code and then uncompress that code into a format that best matches original input data. To obtain the valuable features, the input dimension is a constraint to be greater than the code dimension, which is also known as an undercomplete AE [26]. This kind of learning representation forces the AE to capture the most prominent features of the training data [138]. Formally, the complete encoding and decoding process can be defined as follows:

(2) $$h=\mathrm{encoder}\left(x\right)=g_{e}\left(b_{e}+W_{e}x\right)$$

(3) $$\hat{x}=\mathrm{decoder}\left(h\right)=g_{d}\left(b_{d}+W_{d}h\right)$$

where: h defines the feature vector after encoding; x defines the original input vector; $\hat{x}$ defines the reconstructed input vector; $\left\{b_{e},W_{e}\right\}$ and $\left\{b_{d},W_{d}\right\}$ are the respective biases and weights of encoder and decoder; and $g_{e}$(·) and $g_{d}$(·) are the activation function such. Importantly, AEs may be stacked to develop a deeper network, called a stacked AE, which possesses more encoding layers to extract more abstract representations. Moreover, the AE has the following types: sparse, denoising, contractive, and variational AEs. In the context of virtual sensor design, AEs have been mainly used for addressing the feature selection issue [123,148,149]. The use of DL for selecting the independent parameters is a significant advance in data-driven models since there is currently a lack of a systematized procedure for their selection. For instance, common strategies include principal component analysis, correlation analysis, partial least squares regression, mutual (or partial mutual) information, and many other methods [133]. Recently, Szeląg et al. [146] proposed a weight factor-based method that considers factors such as the number of input parameters and the duration and cost of measuring the quality indicators, among others. 

### 5.2. Deep Belief Networks (DBNs)

DBNs were invented to primarily solve the training problems encountered when using traditional NNs, such as slow learning, requiring a lot of training datasets, and getting stuck in local minima [150]. A DBN is a probabilistic model formed by stacking several restricted Boltzmann machines (RBMs) on top of each other. In the stack, layers can communicate with both the subsequent and previous layers. Apart from the first and last layers, each layer performs the dual role of serving as a hidden layer to incoming nodes and as an input layer to the nodes coming after [151]. The combined distribution between visible layer v and the *l* hidden layers h*^k^* is defined as follows:

(4) $$p=\left(v,h^{1},\ldots ,h^{k}\right)=\overset{l=2}{\underset{k=0}{\prod }}P\left(h^{k}{|h}^{k+1}\right)P\left(h^{l-1},h\right)$$

where: $P\left(h^{k}{|h}^{k+1}\right)$ defines the distribution for the observable units conditioned on the hidden RBM units at level *k*; and $P\left(h^{l-1},h\right)$ defines the visible-hidden combined distribution in the top-level RBM. DBNs are pre-trained through an algorithm called the greedy algorithm. The algorithm utilizes a layer-wise approach for learning essential generative weights. These weights determine the dependence of variables between layers [152]. DBNs address some of the open virtual sensor design problems as follows:

(i) *Labelled data scarcity and the unsupervised feature exploitation*: the DBN’s unsupervised learning phase, followed by a supervised fine-tuning (or semi-supervised learning capability), provides a valuable solution to the labelled data scarcity problem. Additionally, the features extracted during the learning stage can be used for inferring information on the model structure [133];
(ii) *Model complexity reducing*: a DBN is characterized by the utilization of multiple layers of representation which enables the approximation of more complex functions with the reduced number of parameters [133]. This facilitates the model complexity reduction, particularly in terms of the number of model parameters (weights and biases) and the model order.

### 5.3. Convolutional Neural Networks (CNNs)

CNN is a kind of deep NN mainly used for processing data with grid-like topology like image data (2-D grid of pixels) and time-series data (1-D grid taking samples at regular time intervals) [26]. CNNs have three principal components: convolution, pooling, and activation function. The convolution operation can extract features from the data set (through which their spatial information can be conserved), while the pooling operation reduces the feature maps dimensionality from the convolution operation [153]. It is worth noting that the *convolution* in this context defines the cross-correlation function, which is similar to convolution except that the kernel is not flipped [26]:

(5) $$S\left(i,j\right)=\left(I*K\right)\left(i,j\right)=\underset{m}{\sum }\underset{n}{\sum }I\left(i+m,j+n\right)K\left(m,n\right)$$

where: *I* and *K* represent 2-D input and 2-D kernel functions; the character “∗” represents the operation of convolution; and i and j are indexes in the two dimensions. These networks are particularly suited for processing grid-like data because they include sparse interactions, parameter sharing, and equivariant representations [26]. The relevance of CNNs in virtual sensor design is based on the fact that they overcome two limitations of AEs and DBNs. For instance, stacked AE and DBN models are mostly static and therefore do not consider the process data’s dynamic characteristics [142]. Moreover, these models mainly learn the global data features (neglecting the parameters’ dynamics and local correlations) since they are usually fully linked networks [142]. On the other hand, a CNN can extract these dynamic and local features from the data samples by augmenting each sample’s 2-D dynamic matrix, where several lagged input parameters are used to represent the process’s dynamics [143,153].

### 5.4. Echo State Networks (ESNs)

An ESN is a specific type of recurrent neural network (RNN) with powerful modeling capabilities due to the presence of the reserve layer. RNNs are considered a universal approximation method for dynamical systems [139]. They can model the nonlinear dynamical properties found in the noisy industrial data by utilizing a structure with two layers and efficient training: a recurrent nonlinear layer with random fixed weights and a linear adaptive output layer [154]. However, the use of RNNs in practical applications is often constraint by (i) the reliance of the training method on gradient descent, which presents prolonged convergence features, (ii) the existence of bifurcations during training, and (iii) the vanishing gradient [139,154].

Therefore, reservoir computing (RC) was developed to overcome these training problems [155]. A particular type of RC, an ESN, has become a prevalent DL technique due to its ability to capture the dynamic data relationships and the ability to avoid the non-converging and computationally expensive gradient descent method [139]. The following state update equation gives the discrete-time dynamics of the ESN:

(6) $$x\left[n+1\right]=\left(1-\alpha \right)x\left[n\right]+\alpha f\left(W_{r}^{r}x\left[n\right]+W_{i}^{r}u\left[n\right]+W_{o}^{r}y\left[n\right]+W_{b}^{r}\right),$$

and by the output computed as:

(7) $$y\left[n+1\right]=g\left(W_{r}^{o}x\left[n+1\right]+W_{i}^{o}u\left[n\right]+W_{o}^{o}y\left[n\right]+W_{b}^{o}\right)$$

(8) $$=g\left(W^{\mathrm{out}}\left(x\left[n+1\right],u\left[n\right],y\left[n\right],1\right)\right)$$

(9) $$=g\left(W^{\mathrm{out}}z\left[n+1\right]\right)$$

where: α defines the leak rate [154]; f(·) =tanh(·) is the activation function frequently used for ESNs [140]; g defines the post-processing activation function; $W^{\mathrm{out}}$ is the concatenation of $W_{r}^{o}$, $W_{i}^{o}$, $W_{o}^{o}$ and $W_{b}^{o}$; and $z\left[n+1\right]=\left(x\left[n+1\right],u\left[n\right],y\left[n\right],1\right)$ defines the extended reservoir state, and a bias term, respectively. A key issue for ESN is to determine $W^{\mathrm{out}}$ utilizing the known samples [156]. The ESN’s low computational complexity and its ability to capture the process data’s dynamic relationships (due to the reservoir’s self- and feedback connections and a sparse connection weight matrix) makes it ideal for real-time monitoring, fault detection (important for data quality assurance), and monitoring the reliability of the model predictions [139,141]. Monitoring the reliability is achieved by tracking the ESN reservoir’s internal state values. Tracking these values also indicates whether the model needs to be retrained, which can be essential due to the natural evolution of the complex industrial (and environmental) processes [139].

### 5.5. Generative Adversarial Networks (GANs)

The biggest drivers of recent progress in DL have been the availability of massive data, the advancement of modeling architectures, and robust computation. To avoid overfitting, DL models depend on big datasets [157]. Furthermore, adequate training samples can significantly improve the computational speed and prediction performance of virtual sensor modeling procedures [145,158]. Unfortunately, many application domains, including virtual sensor design, do not have access to big datasets [133]. For this reason, generative models are critical for generating simulative or fictive datasets. Of all the types of generative models, the GAN is undeniably the most powerful [159]. GANs consist of two NNs competing against each other. One network, the *generator*, generates synthetic but convincing samples $x=\in p_{fake}$ that represent a real data distribution $p_{data}$. The generator transforms the noise vectors z drawn from $p_{z}$ into new data, i.e., $x=G\left(z\right)$. The other network, the *discriminator*, has access to both $p_{data}$ and data samples generated by G, and attempts to discriminate (or distinguish) between the two [160]. Both networks are trained by solving the optimization problem that the generator is attempting to minimize, and the discriminator is attempting to maximize, as follows:

(10) $$\underset{G}{\min}\;\underset{D}{\max}\;V\left(D,G\right)=\underset{x\sim p_{data}}{E}\left[\log D\left(x\right)\right]+\underset{z\sim p_{z}}{E}\left[\log\left(1-D\left(G\left(z\right)\right)\right)\right]$$

where: *G* refers to the generator, *D* refers to the discriminator, *V*(*D*,*G*) refers to the objective function, $p_{data}$ refers to real samples distribution, and $p_{z}$ refers to the distribution from where the noise vectors are drawn. The discriminator network’s final layer uses a sigmoid activation function [160], so that $D\left(x\right),D\left(G\left(z\right)\right)\in \left[0,1\right]$.

## 6. Conclusions

This study presented a detailed review of the feasibility of virtual sensing for real-time monitoring of surface and groundwater sources for irrigation purposes. Firstly, it introduces readers to surface and groundwater quality crises, including the water quality monitoring evolution from conventional to smart (or internet of things)-based monitoring. Secondly, it discusses the key parameters for monitoring water for irrigation purposes, including developing the qualitative cost model (or estimate) for traditional monitoring of the selected parameters. Thirdly, the review provides a general guideline for virtual sensor design and discusses the fundamentals of virtual sensing, particularly for dummies. Fourthly, the review presents a comprehensive survey of the current status of virtual sensing applications for irrigation water quality assessment. For this aspect, several variables (input and output parameters, predictors pre-processing, data quality, and commonly used techniques) are compared and discussed. Fifthly, it formulates a comprehensive specification book for real-time monitoring of water quality. This specification book presents the global system architecture of virtual sensor monitoring in an internet of things environment and the updated cost model that includes this global system architecture. Finally, the review briefly describes some of the deep learning techniques that can enrich the virtual sensing realization for water quality assessment. These techniques can reduce the computational complexity, aid in learning complex models, efficiently utilize unlabelled data, and can also extract high-level features from data [133]. The study concludes that future research targeting the real-world implementations of smart monitoring for water quality assessment will be essential for increasing the awareness of these advances to the traditional monitoring solutions.

## Figures and Tables

**Table 1:** A list of key water quality parameters monitored for irrigation water.

Anion	Cation	Chemical	Physical	Biological	Heavy Metal
Chloride	Sodium	pH	EC	*E. coli*	Boron
Sulphate	Calcium	DO			
Carbonate	Potassium	COD			
Bicarbonate	Magnesium				
Total nitrogen					

DO = dissolved oxygen; COD = chemical oxygen demand; EC = electrical conductivity; *E. coli* = *Escherichia coli*.

**Table 2:** Water quality indices for irrigation suitability.

Water Quality Index	Symbol	Formula	References
Potential Salinity	PS	$\mathrm{PS}={\mathrm{Cl}}^{-}+\left({\mathrm{SO}}_{4}{}^{2-}\right)/2$	[55,56]
Kelly Index	KI	$\mathrm{KI}={\mathrm{Na}}^{+}/\left({\mathrm{Ca}}^{2+}+{\mathrm{Mg}}^{2+}\right)$	[38,57]
Sodium Adsorption Ratio	SAR	$\mathrm{SAR}={\mathrm{Na}}^{+}/\sqrt{\left({\mathrm{Ca}}^{2+}+{\mathrm{Mg}}^{2+}\right)/2}$	[38,55]
Magnesium Adsorption Ratio	MAR	$\mathrm{MAR}=\left[{\mathrm{Mg}}^{2+}/\left({\mathrm{Ca}}^{2+}+{\mathrm{Mg}}^{2+}\right)\right]\times 100$	[55,57]
Residual Sodium Carbonate	RSC	$\mathrm{RSC}=\left({\mathrm{CO}}_{3}{}^{2-}+{\mathrm{HCO}}_{3}{}^{-}\right)-\left({\mathrm{Ca}}^{2+}+{\mathrm{Mg}}^{2+}\right)$	[38,55]
Exchangeable Sodium Percentage	ESP	$\mathrm{ESP}=\left[{\mathrm{Na}}^{+}/\left({\mathrm{Ca}}^{2+}+{\mathrm{Mg}}^{2+}+{\mathrm{Na}}^{+}+K^{+}\right)\right]\times 100$	[54,55]
Permeability Index	PI	$\mathrm{PI}=\left[\left({\mathrm{Na}}^{+}+\sqrt{{{\mathrm{HCO}}_{3}}^{-}}\right)/\left({\mathrm{Ca}}^{2+}+{\mathrm{Mg}}^{2+}+{\mathrm{Na}}^{+}\right)\right]\times 100$	[55,56]
Sodium Percentage	Na%	$\mathrm{Na}\%=\left[\left({\mathrm{Na}}^{+}+K^{+}\right)/\left({\mathrm{Ca}}^{2+}+{\mathrm{Mg}}^{2+}+{\mathrm{Na}}^{+}+K^{+}\right)\right]\times 100$	[38,56]

**Table 3:** Classification of surface water quality based on water quality indices [38].

Water Quality Index	Range	Water Class
Kelley Index (KI)	<1	Good Unsuitable
	>1	
Sodium Adsorption Ratio (SAR)	<10	Excellent
	10–18	Good/safe
	18–26	Doubtful/moderate
	>26	Unsuitable
Residual Sodium Carbonate (RSC)	<1.25	Good
	1.25–2.5	Doubtful
	>2.5	Unsuitable
Permeability Index (PI)	>75%	Good-Class I
	25–75%	Good-Class II
	<25%	Unsuitable-III
Sodium Percentage (Na%)	<20	Excellent
	20–40	Good
	40–60	Permissible
	60–80	Doubtful
	>80	Unsuitable

**Table 4:** Irrigation water quality specifications [32].

Potential Irrigation Problem	Units	The Degree of Restriction on the Use		
		None	Slight to Moderate	Severe
Electrical conductivity at 25 °C	dS/m	<0.7	0.7–3	>3
Boron	mg/L	<0.7	0.7–3	>3
NO_3_	mg/L	<5	5–30	>30
Chloride	mg/L	<4	4–10	>10
Dissolved oxygen	mg/L	-	-	-
Chemical oxygen demand	mg/L	-	-	-
*Escherichia coli*	cfu/100 mL	-	-	-
pH		Normal range 6.5–8.4		

**Table 5:** An overview of sensor accuracies based on published literature and our proposed accuracy ranges.

Parameter			Accuracy Ratings		
	Target	Acceptable	Tolerable	Poor	Ref.
pH	≤±0.2 units	>±0.2–0.5 units	>±0.5–0.8 units	>±0.8 units	[62,63,64,65]
Electrical conductivity	≤±3%	>±3–10%	>±10–15%	>±15%	[61,62,63,64]
Dissolved oxygen	≤±5%	>±5–10%	>±10–15%	>±15%	[61,62,63,64,65]
Total nitrogen	≤±5%thisreview [TR]	>±5–10% [63,64]	>±10–15%	>±15%	[TR]
Chloride	≤±5%	>±5–10% [64]	>±10–15% [63]	>±15%	[TR]
Calcium	≤±5%	>±5–10% [64]	>±10–15%	>±15%	[TR]
Sodium	≤±5% [65]	>±5–10%	>±10–15%	>±15%	[TR]
Chemical oxygen demand	≤±5% [67]	>±5–10% [65]	>±10–15%	>±15%	[TR]
Boron	≤±5%	>±5–10%	>±10–15%	>±15%	[TR]
Sulphate	≤±5%	>±5–10%	>±10–15%	>±15%	[TR]
Potassium	≤±5%	>±5–10%	>±10–15%	>±15%	[TR]
Alkalinity	≤±5%	>±5–10%	>±10–15%	>±15%	[TR]
Magnesium	≤±5%	>±5–10%	>±10–15%	>±15%	[TR]
*Escherichia coli*	≤±5%	>±5–10%	>±10–15%	>±15%	[TR]

**Table 6:** Cost estimate per parameter.

Monitoring Activity	In Situ Measurement							Laboratory Analysis						
	pH	EC	DO	Na	Ca	Mg	Cl	SO_4_^2^	K	B	Alkal	TN	COD	*E. coli*
Sample preservation	0.0	0.0	0.0	0.0	0.0	0.0	0.0	0.5	0.5	0.0	0.0	0.5	0.5	1.0
Transportation cost	0.0	0.0	0.0	1.0	1.0	1.0	1.0	1.0	1.0	1.0	1.0	1.0	1.0	1.0
Labour	0.5	0.5	0.5	1.0	1.0	1.0	1.0	1.0	1.0	1.0	1.0	1.0	1.0	1.0
Equipment costs	0.0	0.0	0.0	1.0	1.0	1.0	1.0	1.0	1.0	1.0	1.0	1.0	1.0	1.0
Consumables	0.0	0.0	0.0	0.5	0.5	0.5	0.5	0.5	0.5	1.0	0.5	1.0	1.0	0.5
Measurement duration	0.0	0.0	0.0	0.0	0.0	0.0	0.0	0.0	0.0	0.5	1.0	0.5	1.0	1.5
Communication + computing	1.0	1.0	1.0	1.0	1.0	1.0	1.0	1.0	1.0	1.0	1.0	1.0	1.0	1.0
**Total score**	**1.5**	**1.5**	**1.5**	**4.5**	**4.5**	**4.5**	**4.5**	**5.0**	**5.0**	**5.5**	**5.5**	**6.0**	**6.5**	**7.0**
**Cost estimate**	**L**	**L**	**L**	**H**	**H**	**H**	**H**	**H**	**H**	**H**	**H**	**H**	**VH**	**VH**

EC = electrical conductivity; DO = dissolved oxygen; Alkal = alkalinity; TN = total nitrogen; COD = chemical oxygen demand; *E. coli* = *Escherichia coli*; L = low; H = high; VR = very high.

**Table 7:** Details of the studies reviewed.

Inputs ^1^	Pre-Processed	Outputs ^2^	Data Time Scale	Techniques ^3^	Ref.
EC, pH, TD, fDOM, HP, Temp, Turb, SM	No	TP, TN	2018–2019 (1–15 min)	RF	[94]
DO, Turb, pH, Temp, ORP, EC	Yes	BOD	February–April 2019 (NS)	MLR, MLP, SVM-SMO, IBK, RF	[29]
EC, Temp, pH	Yes	TDS, PS, SAR, ESP, MAR, RSC	2009–2019 (NS)	ANN, MLR, RF, SVM, kNN, Adaboost	[55]
EC, pH	Yes	SAR, ESP, %Na, RSC, PI, KI, Cl, MAR, TDS	NS (NS)	ANN, MLR, RF, SVM, kNN, Adaboost	[95]
DO, Temp, TSS, N^−^, h NH_3_, pH, TOC, Turb	Yes	COD	NS (NS)	MLR, MLP, SVM, RF, kNN	[22]
EC, Turb, Temp, DO, pH, Chl-a, Q	Yes	TP, TN	2009–2012 (hourly)	RF	[85]
TSS, TDS, Turb, EC, COD, BOD	No	TP, TN	2009–2014 (bimonthly)	RF, MLR	[96]
NS	NS	BOD, DO	NS (NS)	NM	[97]
Temp, NH_4_-N, DO, DLS, pH	Yes	WQ	January 2010–December 2012 (monthly)	FNN, HK-FNN	[98]
EC, pH, TDS, Ca, K, CO_3_, Na, Mg, HCO_3_, Cl, SO_4_	No	SAR	1971–2017 (NS)	RF, GMDH	[99]
NS	NS	Eutrophication	NS (NS)	NM	[100]
Turb, OP, Chl-a, Cl	No	TP	NS (monthly)	MLR	[101]
EC, pH, Na, Ca, Mg, K, CO_3_, HCO_3_, NO_3,_	No	SAR, RSC, MAR, KI, %Na, SO_4_, TDS, TH, Cl	2012 (NS)	ANN	[102]
Temp, pH, DO, EC, TN	Yes	Algal blooms	May 2010–August 2010 (6 times/hour)	GPR, MLP, BNN, MLR	[103]
Turb, Temp, pH, EC	No	TP	April 2010–September 2013 (hourly)	MLR	[104]
Turb, Temp, DY, HD, SS, SE	No	TP, TSS	August 2005–April 2008 (30 min)	MLR	[105]

^1^ TD = time of day; fDOM = fluorescence dissolved organic matter; HP = hydrostatic pressure; Temp = temperature; Turb = turbidity; SM = soil moisture; ORP = oxidation reduction potential; TSS = total suspended solids; h = water level; TOC = total organic carbon; Chl-a = chlorophyl-a; Q = flow rate; TDS = total dissolved solids; BOD = biological oxygen demand; NS = not specified; DLS = NS; OP = orthophosphorus, TH = total hardness; DY = day of year; HD = hour of day; SS = spring snowmelt; SE = storm event; ^2^ TP = total phosphorus; ^3^ RF = random forest; MLR = multi linear regression; MLP = multilayer perceptron; SVM-SMO = support vector machine-sequential minimal optimization; IBK = instance-based learner; ANN = artificial neural network; kNN = k-nearest neighbour; Adaboost = adaptive boosting; NM = numerical model; FNN = fuzzy neural network; HK-FNN = hierarchical clustering and k-means algorithm-fuzzy neural network; GMDH = group method of data handling; BNN = Bayesian neural network.

**Table 8:** An updated cost estimate per parameter.

Monitoring Activity	In Situ Measurement			Laboratory Analysis										
	pH	EC	DO	Na	Ca	Mg	Cl^−^	SO_4_^2−^	K	B	Alkal	TN	COD	*E. coli*
Sample preservation	0	0	0	0	0	0	0	0.5	0.5	0	0	0	0	0
Transportation cost	0	0	0	0	0	0	1	1	1	1	0	0	0	0
Labour	0.5	0.5	0.5	0	0	0	1	1	1	1	0	0	0	0
Equipment costs	0	0	0	0	0	0	1	1	1	1	0	0	0	0
Consumables	0	0	0	0	0	0	0.5	0.5	0.5	1	0	0	0	0
Measurement duration	0	0	0	0	0	0	0	0	0	0.5	0	0	0	0
Communication + computing	1	1	1	1	1	1	1	1	1	1	1	1	1	1
**Total score**	**1.5**	**1.5**	**1.5**	**1**	**1**	**1**	**4.5**	**5**	**5**	**5.5**	**1**	**1**	**1**	**1**
**Cost estimate**	**L**	**L**	**L**	**L**	**L**	**L**	**H**	**H**	**H**	**H**	**L**	**L**	**L**	**L**

EC = electrical conductivity; DO = dissolved oxygen; Alkal = alkalinity; TN = total nitrogen; COD = chemical oxygen demand; *E. coli* = *Escherichia coli*; L = low; H = high.

**Table 9:** Target accuracies for the five high-frequency measurable parameters.

Parameter	Target Accuracy	Reference
pH	≤±0.2 units	[62,63,64,65]
Turbidity	≤±5%	[63,64,65,66,67]
Temperature	≤±0.5 °C	[63,64,65,66,67]
Conductivity	≤±3%	[61,62,63,64]
Dissolved oxygen	≤±5%	[61,62,63,64,65]
